# Work from Home Success: Agile work characteristics and the Mediating Effect of supportive HRM

**DOI:** 10.1007/s11846-022-00545-5

**Published:** 2022-04-12

**Authors:** Lukas Heidt, Felix Gauger, Andreas Pfnür

**Affiliations:** Department of Real Estate Management and Construction Management, Technical University of Darmstadt, Hochschulstraße 1, 64289 Darmstadt, Germany

**Keywords:** Agile workforce, Telework, Work from Home, Dynamic capabilities, HRM, Firm success, M10, M12, M14, M59, D22, D23

## Abstract

Work from home or teleworking, continues to expand not least due to the COVID-19-crisis and poses challenges for employees and companies. In uncertain and dynamic times, organisations wonder what skills make employees successful when working from home and which measures support employees. By performing in-depth research that addresses employee agility as skills and capabilities, a research framework is proposed. Based on an international survey of employees working from home during the COVID-19-crisis (N = 1,016), the impact of agile work characteristics on work from home success and the mediating effect (accounting for 48% of the total effect) of tailored support measures by HRM were investigated. The results of the mediation analysis show that agile work characteristics have a direct, positive and significant effect on the success of working from home. Part of the effect is explained by HRM measures as a mediator. The findings contribute to the research stream of dynamic capabilities by applying the theory to working from home. The comparatively simple research model provides companies with information on how they can best support employees in the dynamics of a crisis and the expansion of work from home and, therefore, has high relevance for practitioners.

**Supplementary Information:**

The online version contains supplementary material available at 10.1007/s11846-022-00545-5.

## Introduction

Companies are subject to constant change and must adapt in order to survive. The success of a company depends on how business and human resource management (HRM) can respond to changes in the work environment. A growing shift towards work from home (WFH), or telework, with potential benefits such as employee retention, job satisfaction, higher employee productivity and enhancement of overall organisational performance, can be observed (Bloom et al. 2015; Fonner and Roloff 2010; Martin and MacDonnell 2012; Martínez-Sánchez et al. 2007b). Meanwhile, among the drivers of WFH is another factor: the economic and social crisis caused by COVID-19 (Abulibdeh 2020; Belzunegui-Eraso and Erro-Garcés 2020). Due to this pandemic, the widespread use and in some cases massive expansion of WFH became necessary, and it is to be expected to continue long after the COVID-19-pandemic (Brynjolfsson et al. 2020). 42% of the U.S. labour force is working from home due to the pandemic. This accounts for more than two-thirds of U.S. gross domestic product based on their earnings (Bloom 2020).

The expansion of WFH represents a change for employees and companies with challenges such as altered leadership models, distance from colleagues and more difficult communication and collaboration (Caligiuri et al. 2020; Contreras et al. 2020; Raišienė et al. 2020; Smith et al. 2018). Employees with low tolerance for uncertainty can be negatively affected, which may lead to distress and reduced well-being (Smith et al. 2020). Management, and especially HRM, must therefore find ways to support employees facing change and uncertain situations while working from home and, thereby, ensure the success of the company (Gigauri 2020). Without appropriate support for these challenges, employees are potentially affected by loneliness or loss of meaning and companies are threatened in their ability to survive (Carnevale and Hatak 2020; Tremblay and Thomsin 2012).

The benefits of WFH for companies and employees have been studied, especially in terms of work equipment, productivity, work–life balance, flexible time allocation and reduced time loss due to less commuting (Gálvez et al. 2011; Nakrošienė et al. 2019). However, surprisingly few studies exist that examine what skills and organisational conditions make employees successful in working from home (Turetken et al. 2011). While there is work that relates employee characteristics to firm success, particularly in terms of flexibility (Beltrán-Martín et al. 2008; Bhattacharya et al. 2005), there has been little application of these concepts to WFH.

In this context, the question arises, especially in times of crisis, as to which actions HRM must take to guarantee success when working from home. Enhanced flexibility or employee autonomy could be goals for HRM in this regard (Fenton-O’Creevy et al. 2008). In times of high uncertainty, such as COVID-19, it is even more crucial for HRM to support the organisation to deal with change and achieve the resilience of employees to uncertainty by facilitating dynamic capabilities (Batra 2020; Carnevale and Hatak 2020).

Various approaches and theories in the field of dynamic capabilities exist that deal with the capabilities of a company and its employees to dynamically react and adapt to changes in an uncertain environment (e.g. Overby et al. 2006, Li and Liu 2014 or Tallon et al. 2019). Some of these have already been described as beneficial in the context of the COVID-19-crisis (Janssen and van der Voort 2020) but have not yet been applied to WFH settings. This ability to adapt to unpredictable circumstances and rapid changes can be described as ‘agility’ (Overby et al. 2006). Agility builds on theories that deal with adaptability, perception and reaction to change, and with associated competitive advantages. In this context, agility is a construct with diverse definitions and its role in business survival has additionally increased during the COVID-19-crisis (Werder et al. 2020).

In particular, the work and attitudes of employees play a central role in achieving an agile organisation (Muduli and Pandya 2018), which is also called an ‘agile workforce’ (Breu et al. 2002; Samukadas and Sawhney 2004; Sherehiy et al. 2007). This includes individual employee skills and behaviours (Muduli 2013) as well as appropriate agile work design characteristics (Sherehiy et al. 2007), culture (Felipe et al. 2017) and leadership (Parker et al. 2015; Uhl-Bien and Arena 2018). Agile capabilities and behaviours of employees are, for example: participatory solution finding, willingness to change and adapt, tolerance of uncertainty, finding solutions through teamwork, or coping with challenging work through autonomy (Dyer and Shafer 2003; Griffin and Hesketh 2003; Samukadas and Sawhney 2004). Most studies in this field identify the benefits of agile employee and organizational capabilities in terms of uncertainty and adaptability but are mostly related to traditional work environments and not to WFH. Other researchers highlight the fact that flexibility is a possible facilitator for WFH as a work model (Martínez-Sánchez et al. 2007b; Martínez-Sánchez et al. 2008). However, the adaptation of agile or flexible skills and organisational forms into WFH has not yet been researched, or is limited to selected characteristics (e.g. Turetken et al. 2011).

In addition, studies show that employees need support to work optimally from home. This includes formal and informal communication, training, supervisor support, appropriate technology and social interactions (Berube Kowalski and Swanson 2005; Greer and Payne 2014). HRM designed for WFH is critical in this regard to promote autonomy and personal responsibility (Martínez-Sánchez et al. 2008). In the case of changes in general and WFH in particular, HRM must accompany changes accordingly (Heidt et al. 2020). HRM should, therefore, encourage the participation of employees and integrate them directly into the change process.

Studies have demonstrated the impact of HRM measures that support WFH on organisational success (Martínez-Sánchez et al. 2007b). However, there is a lack of studies examining whether employees in agile organisations require special or less pronounced support from HRM. For one, employees in an agile workforce are autonomous and adaptive, yet agility and associated characteristics, such as skill variance, are also complex and challenging (Sherehiy and Karwowski 2014). Therefore, we analyse if the enablement of employees working from home has a mediating effect. In doing so, information is provided on which special measures are necessary to support agile employees working from home.

Based on literature streams of WFH and agile work, our study makes two contributions. First, we analyse existing models of employee and organisational agility grounded on the dynamic capability theory (Breu et al. 2002; Muduli 2016; Overby et al. 2006; Tallon et al. 2019; Teece et al. 2016).

We propose an adapted model of agile work with ten characteristics from the field of dynamic capabilities by transferring the work of Sherehiy and Karwowski (2014) to the special context of WFH. In this way, we expand the understanding of success factors of WFH with a focus on characteristics of employees and organisation, which have only a subordinate role in current research (Baruch 2001; Turetken et al. 2011).

Second, we add to literature in the area of appropriate support for WFH by showing that there are support measures like HRM support that directly increase WFH employees’ success while at the same time also indirectly contribute to employee success by mediating the effect of employee characteristics such as agile work (Martínez-Sánchez et al. 2007b; Turetken et al. 2011; Uhl-Bien and Arena 2018). Thus, we contribute to organisational and HRM literature by combining the strands of workplace and dynamic capabilities literature, and give practical and theoretical implications. This quantitative work provides insights into the mechanisms of various factors and their relationships on WFH success, and opportunities for optimised deployment in dynamic environments even after COVID-19.

## Theoretical framework and hypotheses

According to the resource-based theory of management, the success of a company is essentially attributed to its capabilities and resources (Wernerfelt 1984). These capabilities, which are difficult for competitors to copy, can be described as the tangible or intangible resources, skills or knowledge of individuals. In this respect, the workforce and employees, with their individual knowledge, experience and skills, are among the most important resources of an organisation (Coff 1997). These resources and capabilities can also include so-called *dynamic capabilities* as the ability to act successfully in uncertain environments (Teece et al. 1997). Dynamic capabilities represent a construct that refers to the entire enterprise and deals with actions in a rapidly changing environment. In this context, dynamic capabilities are a crucial competitive advantage in uncertain environments and, therefore, are not only a moderator of success but a central driver (Li and Liu 2014). A central idea here is the maintenance of competitiveness through adaptability (Overby et al. 2006). Various concepts exist that can be attributed to dynamic capabilities and their achievement: enterprise agility (Overby et al. 2006; Sherehiy et al. 2007), organizational agility (Côrte-Real et al. 2017; Nijssen and Paauwe 2012; Tallon et al. 2019), and the general, often synonymously used term, ‘flexibility’, for agility in this context (Teece et al. 2016). However, this should not be confused with agility in the sense of, for example, agile project management or agile software development even though these have similar origins (Janssen and van der Voort 2020). Adaptations of these concepts with a focus on employees and their work extend the goals of dynamic capabilities to the workforce as part of a company’s resources. Employee adaptability (van Dam 2013), human resource flexibility (Bhattacharya et al. 2005; Martínez-Sánchez et al. 2007a), agile workforce (Alavi et al. 2014; Breu et al. 2002; Muduli 2013; Sherehiy and Karwowski 2014) and workforce scalability (Nijssen and Paauwe 2012) are possible concepts because they enable flexible and adaptive actions in dynamic environments. This paper summarises these concepts under the term a*gile work*.

**Fig. 1:**
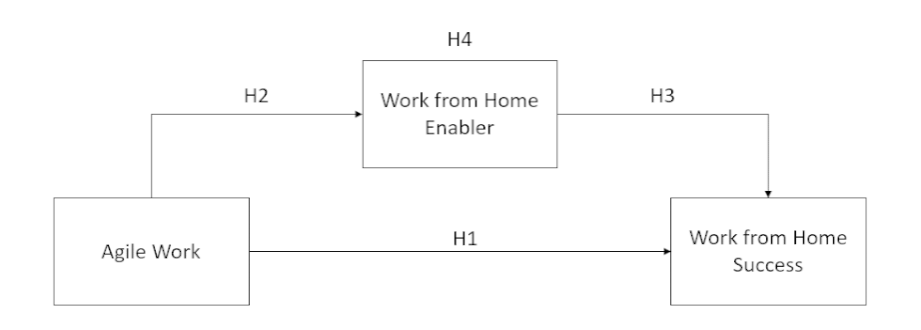
Conceptual model - agile work and the relationship to WFH success

The framework proposed in this paper includes three constructs: agile work, WFH enabler and WFH success (Fig. 1). Agile work summarises the pronounced agility of employees (Sherehiy and Karwowski 2014). WFH enabler summarises the extent to which expected organisational support services are provided for WFH (Berube Kowalski and Swanson 2005). WFH success measures productivity and satisfaction of WFH at employee-level (Baruch 2001). These concepts are not measured at the firm level but at the self-assessment individual level as employees are the key reference for this study (Shin et al. 2000).

This conceptualisation of possible impacts on WFH success through a rather simple model differs from similar approaches. Unlike, for example, Martínez-Sánchez et al. (2007a) or Smith et al. (2020), a less complex model was deliberately chosen; simple models may generalise better and additional independent variables, for example, do not equate to higher accuracy or explanatory power (Dul and Ceylan 2014). To date, only few studies exist that link employee skills related to agility with WFH. Compared with the more complex structural equation model (e.g. Alavi et al. 2014), and particularly because the research is still in its infancy, a simple model focusing on agile work and enablers is particularly suitable to model the direct and mediated effects.

Figure 1 shows the conceptual model. Agile work is related to WFH success for several reasons. The autonomy of WFH does not suit every individual and can have negative effects. Furthermore, WFH can be described as ‘demanding’ because different skills, more personal responsibility and proactivity are needed when working from home (Turetken et al. 2011). This is also part of agile work and, thus, can be beneficial in this work setting. Working independently can accommodate agile employees because they value autonomy, flexible job design and job control (Sherehiy and Karwowski 2014; Turetken et al. 2011). Agile work is characterised by active bidirectional communication, proactivity and shared team responsibilities (Hopp and Oyen 2004; Muduli 2013). Employees can communicate proactively or maintain team bonding and, at the same time, have freedom in planning their work. This results in fewer interruptions while maintaining an exchange of information despite distance to colleagues, which leads to an increase in productivity (Fonner and Roloff 2010). Increasing uncertainty and dynamism in the COVID-19-pandemic are amplified in a WFH setting (Carnevale and Hatak 2020). On the one hand, agile work is associated with adaptivity to change. This allows short-term changes to be considered as part of the work. Likewise, a flexible style of WFH helps implement changes. Therefore, we propose that the WFH setting suits agile employees and enhances WFH success. Thus, a possible positive effect of agile work on WFH success follows:

### Hypothesis 1

(H1): A higher degree of agile work is related with higher WFH success.

However, agile work emphasises skill variance and high job demands (Sherehiy and Karwowski 2014), which may differ from other types of work and can, therefore, be challenging for employees not familiar with these work demands. This makes organisational support advisable. Enablers for WFH can be technical prerequisites as well as technical support and training, an appropriate leadership style like management by objectives, an enabling culture and trust from supervisors, offered participation and the opportunity to design and carry out work in an autonomous manner (Berube Kowalski and Swanson 2005; Martínez-Sánchez et al. 2007b; Nakrošienė et al. 2019). However, it can also be argued that agile employees are not only particularly dependent on support, but also more receptive. An example could be autonomy, which is described positively as a supportive measure but also as an aspect of agile work (Gálvez et al. 2011). Agile work employees rely on management by objectives from superiors, attach importance to being involved in decision-making processes and want to actively participate in them. Other studies emphasise the need for an ‘enabling leadership style’ in dynamic environments (Uhl-Bien and Arena 2018) that supports adaptivity and is often found within employees of agile work. It can, therefore, be assumed that employees in the sense of agile work are more receptive to support and, thereby, have a positive effect on the actions in the sense of WFH enablers:

### Hypothesis 2

(H2): The more pronounced agile work is among employees, the more effectively WFH enablers can be used.

When companies adequately support their employees, it is commonly assumed that this has a positive effect on their performance. Established concepts such as ‘perceived organizational support’ (Eisenberger et al. 2001) focus strongly on perception. However, it can be argued that perceptions of support measures change due to isolation and distance in the WFH and that some measures are particularly important and expected by employees (Gálvez et al. 2011). Employees are separated socially and technically as well as administratively. In this respect, support is essential: without a functioning infrastructure, work is severely hindered (Mello 2007; Morgan 2004). The employee hardly has a chance to get immediate help and is dependent on (expert) support. Furthermore, mentoring, leadership, controlling, and face-to-face communication by superiors are limited due to physical distance (Greer and Payne 2014). Isolation or lack of management commitment are some of the biggest barriers to successful WFH (Mello 2007). Additionally, the manager must find ways to encourage and promote trust and social integration accordingly (Nakrošienė et al. 2019). Thus, there is a potential positive effect of support interventions that target WFH challenges:

### Hypothesis 3

(H3): Employee support is positively associated with WFH success.

In addition to the positive effect of WFH enablers on WFH success described in other studies in a similar manner (e.g. ‘trust’ by Berube Kowalski and Swanson 2005), another effect can be assumed. Due to the characteristics of agile employees, enablers could be used more effectively, are more accepted and valued (H2) and, thus, positively influence WFH success (H3). This means that the positive effect of agile work on WFH success is partly or entirely influenced and mediated by those enablers. According to H1, agile work has potential benefits in application to WFH. However, it can be conjectured that these benefits do not exclusively impact directly but are incorporated via the application of enablers. Based on the definition of the constructs, it is reasonable to assume that there is no full mediation but only partial mediation. Complete mediation would imply that agile work is helpful solely due to or in combination with enablers, but this seems unlikely. Even completely without the defined enablers, it should still be possible to find a positive impact of agile work. We therefore conclude the previous hypotheses and postulate:

### Hypothesis 4

(H4): The use of WFH enablers partially mediates the effect of agile work on WFH success.

In summary, Fig. 1 suggests that agile work could have a general effect on WFH success. We assume that this effect could be mediated using WFH enablers by exploiting the characteristics of agile work.

## Methodology

### Sample and procedure

A quantitative survey was conducted, mainly consisting of closed questions, seven-point Likert scales and some open questions. The respondents were employees who worked from home during the COVID-19-pandemic. The independent variables were collected in two waves. The dependent variable was collected in a separate third wave with the same participants in order to avoid methodological bias (common method bias). In addition, information on support measures was collected in the third wave because a free-text evaluation revealed missing items. Participants completed the first survey from 18 to 22 June, the second survey from 10 to 14 August and the third survey from 8 to 15 October, 2020. The average age of participants was 36.82 years with a standard deviation of 10.47 and ranging from 18 to 70 years. All three questionnaires were responded by the same 1,016 participants: 467 responses from Germany and 549 from the United States. In the first survey wave, 2,415 respondents answered the survey, resulting in a drop-out rate of 57.9%. Further demographic information can be found in Table 1.

### Data collection

In order for findings to be obtained for the purposes of this study, participants had to work at home at least part of the time in order to qualify for the study. We focus on Germany as the European country with the highest GDP and stable economic and political regulations. In order to compare the results with the Anglo-Saxon culture, we selected the U.S. as a country with high GDP, but with differences in COVID-19 handling. Furthermore, WFH is more widespread in the U.S. than in Germany (WFH while the COVID-19 crisis; first wave: U.S. 42% (Bloom 2020); Germany: 27% (Statista 2021). Furthermore, the housing situation in the U.S. is significantly different than in Germany. American citizens tend to have more housing space and rooms available than Germans. At the same time, American office buildings tend to perform worse than German offices (Gauger F., Bachtal Y., Pfnür A. 2022). We recruited German participants via Clickworker and U.S. participants via Amazon’s Mechanical Turk (MTurk). The survey was administrated in Lamapoll and then spread through Clickworker and MTurk, which are sampling platforms with increasing popularity in research to generate fast and reliable respondents. Previous research has proven comparable quality responses to those obtained from more traditional sampling methods (Brawley and Pury 2016; Follmer et al. 2017; Lutz 2016).

Twenty-eight respondents were excluded from the sample because their response time was too short. To address the potential for common method bias, we checked characteristics of the respondents with those of the original population sample of office workers. We found no differences for age, gender and tenure, suggesting that there is no nonresponse bias. Furthermore, we conducted Harman’s single-factor test to check for common method bias. The single factor accounts for 43% (< 50%) of variance, suggesting there is no indication for such a problem (Podsakoff et al. 2012). Respondents are from all different industries (public management service, service, industry/production, retail) with a high proportion from the service industries (38.3%). This might be due to the affinity of respondents from the IT, creative and freelancer sectors towards WFH, which are all clustered under ‘service industry’. In addition, there is a tendency for MTurk to more strongly target technology-related industries, which explains, in combination with the precondition that WFH is possible, the relatively high share of service industries (Keith and Harms 2016).

**Table 1:** Demographics and descriptive statistics of participants (*N* = 1,016)

		M	S.D.
Age (years)		36.82	10.47
Work experience (years)		12.00	9.78
Workhours per week (h)		36.45	11.90
		Frequency	%
Working from home			
Yes		234	23.0
Partially		782	77.0
Gender			
Female (= 2)		378	37.2
Male (= 1)		635	62.5
Other		3	0.29
Country			
Germany		467	47.0
US		549	53.0
Leadership responsibility			
Yes		503	49.5
No		513	50.5
Industries			
Public management service		270	23.3
Service		444	38.3
Industry/Production		207	17.9
Logistics		38	3.3
Retail		55	4.7
Own workroom			
Yes		620	61.0
No		396	39.0

### Measures

#### Agile work (independent variable)

**Fig. 2:**
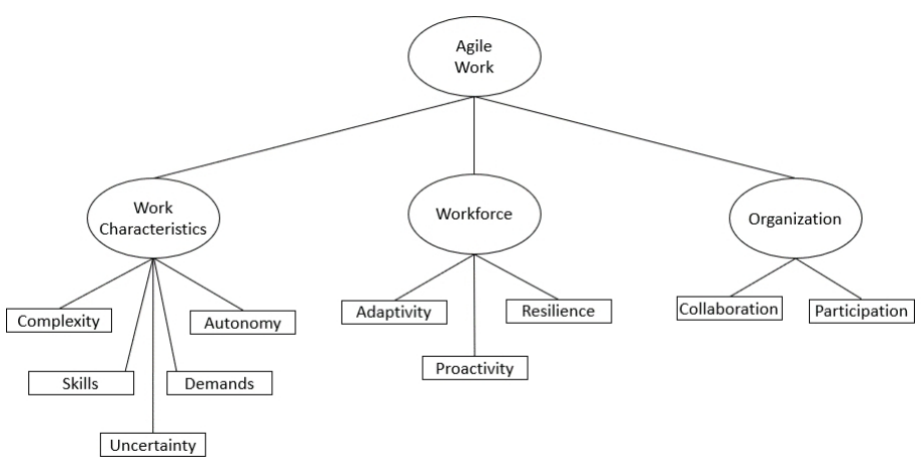
Construction of agile work. Adapted from Sherehiy and Karwowski (2014)

Agile work is a concept that encompasses all the characteristics, attitudes and skills of employees that enable them to work flexibly and adaptably in uncertainty. To define the construct ‘agile work’, relevant studies (including Alavi et al. 2014; Breu et al. 2002; Sherehiy et al. 2007; Sherehiy and Karwowski 2014) were analysed and the dimensions proposed by Sherehiy and Karwowski (2014) were adapted and adjusted with respect to the particular environment of WFH (Fig. 2). This results in three constructs of agile work, which is an adapted scale of the agile workforce scale by Breu et al. (2002) and Muduli and Pandya (2018).

*Work characteristics*: Through an in-depth analysis of studies in the area of work characteristics in agile environments, five characteristics were selected that can positively influence work success in dynamic environments (Bond and Bunce 2003; Breu et al. 2002; Muduli 2017; Sherehiy and Karwowski 2014): *Complexity* describes how demanding a job performed is (e.g. ‘In my job, I have to handle a variety of tasks’); *Skills* describes the extent to which diverse and changing skills are required to perform the job (e.g. ‘In my job, I can use many of my talents’); *Uncertainty* describes the extent to which the employee is confronted with uncertainty with regard to changing goals, work conditions or requirements (e.g. ‘In my work, I am always doing something new’); *Demands* describes the extent to which employees are mentally challenged in performing the job (e.g. ‘My job requires the use of demanding skills’); and *Autonomy* describes the extent to which employees can decide on the sequence, focus and content of the activity (e.g. ‘I can decide for myself the sequence in which I do my work’). The overall Cronbach’s alpha (CRA) of the construct work characteristics is 0.921, which is considered to be sufficiently high.

##### Workforce

‘Agile workforce’ according to Sherehiy and Karwowski (2014) describes skills and behaviours of employees. Three characteristics of a workforce have been the topic in several studies (Alavi et al. 2014; Breu et al. 2002; Muduli 2017; Sherehiy and Karwowski 2014). *Proactivity* refers to a person’s ability to actively take action that will have a positive impact in a changing environment (Sherehiy and Karwowski 2014). Taking initiative and improvisation in the context of uncertainty can be counted among these (e.g. ‘I think about how things might change in the future and try to influence those things in my daily actions’) (Dyer and Shafer 2003). *Adaptivity*, according to Griffin and Hesketh (2003), is the ability to reflect on one’s behaviours and be able to adjust based on a change. Furthermore, this characteristic comes into play when working in roles with various and changing demands (e.g. ‘Short-term changes and adjustments to goal of work are important parts of work for me’) (Sherehiy and Karwowski 2014). *Resilience* describes the ability to perform optimally despite changing conditions and associated challenges. It therefore expresses an independence from challenging situations or failure that would otherwise lead to negative effects such as stress or frustration (e.g. ‘I advocate an error-tolerant culture that does not punish failure but views it as a gain in knowledge’) (Sherehiy and Karwowski 2014). The CRA of workforce has a value of 0.836, which is sufficiently high.

##### Organisation

Through an analysis of studies in the area of higher-level characteristics of an agile organisation, two characteristics were identified that influence employees and their work in uncertain environments (Breu et al. 2002; Muduli 2017). The first dimension concerns *collaboration* between employees. Shared responsibility and decision-making within the team, good cooperation and motivation derived from the team can be attributed to this characteristic (Breu et al. 2002). In particular, shared responsibility is important in uncertainty (e.g. ‘A shared perceived responsibility for work results in the team is important to me’). *Participation* expresses an involvement in decisions that affect one’s situation. Those who actively participate are better able to cope with uncertainty (Muduli 2017). This is sometimes called ‘empowerment’ but it should not be confused with autonomy, the flexible shaping of work and content (e.g. ‘I expect decisions to be made in the team if possible’). The internal consistency of this construct is also confirmed with a CRA of 0.829.

#### Work from home enabler (mediator)

From the multitude of support measures for WFH described in the literature, we focus on two constructs that have particular potential in the context of agile work, or are absolute foundations for successful WFH: *(1) technical infrastructure and support* and *(2) HRM measure*s. Technical infrastructure and support measures taken by the organisation to create all technical prerequisites for WFH can lead to enhanced performance. This refers to information technology measures as well as technical support for employees (Morgan 2004). Employees working from home depend on appropriate technical foundations to perform their agile work (‘I do not expect any technical problems (software, hardware, etc.) when transitioning to the home office’). HRM measures support WFH employees through appropriate processes and culture. Trust between employees and supervisors is the basis for other measures like management by objectives, offering autonomy, opportunity to take responsibility, opportunities to plan and design work and is, therefore, essential as an HRM measure (e.g. ‘In cooperation and in the relationship with superiors, I expect mutual trust’) (Berube Kowalski and Swanson 2005; Martínez-Sánchez et al. 2007b; Nakrošienė et al. 2019). Additionally, regardless of whether change is triggered by an increase of WFH or by changes due to a dynamic environment, measures directed against possible, negative effects of changes like participation have to take place especially in WFH (e.g. ‘I expect to be sufficiently involved in the planning process of increased WFH on the part of my employer’) (Martínez-Sánchez et al. 2008). The CRA for this construct has a value of 0.581, which is slightly below the threshold.

#### Work from home success (dependant variable)

WFH success is measured by *job satisfaction at home* (Fonner and Roloff 2010) and *work productivity at home* (Gamal Aboelmaged and Mohamed El Subbaugh 2012). The three-item ‘Job Diagnostic Survey’ by Hackman and Oldham (1975) was modified to measure job satisfaction at home. Work productivity at home was measured by self-assessment on a modified scale (including, e.g. ‘Working in a home office makes it easier for me to do my job’) (Bloom et al. 2015). The CRA of 0.890 is considered to be sufficiently high.

#### Control variables

To rule out confounding effects, we applied control variables in our analysis. Because other studies have found that openness and affinity for digital technologies may reduce stress due to technology and, therefore, enhance WFH success, we controlled for openness to technology (Suh and Lee 2017). The openness to technology construct consists of 18 items and provides good reliability with a CRA of 0.972. We also controlled for age and gender, according to Bloom et al. (2015). Based on other studies on WFH, we further controlled for a dedicated room for WFH (‘own workroom’; dummy variable). Having an own workroom for WFH can reduce stress and interruptions leading to a higher WFH success. We further controlled for leadership responsibility since perception and demands of WFH may differ between employees with and without leadership (‘leadership’; dummy variable) (Møller-Jensen et al. 2008). To explore if there is a locational effect and a significant difference between the Anglo-Saxon and European work culture, we controlled for the possible effect of the country in which the WFH is done. We thus included a country dummy variable (Germany and U.S. = reference group).

The survey items, descriptive statistics and CRA measures are shown in Table 2, with higher means representing greater levels of each variable. Except the construct WFH enabler (five items), all constructs have Cronbach’s alpha (α) values above the threshold of 0.70 (Cortina 1993).^1^

#### Subsamples

In order to gain further insights, we applied two subgroup analyses. For this purpose, the entirety of the data was subdivided to compare them with each other. The investigated model could provide different results for subgroups of certain characteristics. First, possible differences in outcomes between employees with different work experience (low: < 10 years of work experience, *N* = 287; high: ≥ 10 years of work experience, *N* = 341) were investigated (Torten et al. 2016). There could be different needs and effects of enablers due to different levels of work experience (Puyod and Charoensukmongkol 2019), or agile working could be difficult for inexperienced employees to use, as connected skills are described as demanding (Sherehiy and Karwowski 2014). We thus hypothesise that it might be possible that more experienced employees make better use of agile work and need less support by HRM etc. as they are already able to cope with that high degree of agility and demanding skills.

Second, we examined possible differences between part-time (< 30 h per week, *N* = 123) and full-time employees (≥ 30 h per week, *N* = 505). Part-time employees can respond differently to work attributes and can have differing work satisfaction and success due to their reduced hours as they are less included. Part-time employees need a more defined setting because they are less involved in the workforce so they need more direction, leadership or guidance. Additional autonomy could lead to further distance and isolation and outweigh the benefits. We therefore postulate that there might be a difference between the two subsamples and that part-time employees are not able to benefit from agile work in such a pronounced way (Conway and Briner 2002). Literature further reports that part-time employees are more aware of organisational support than full-time employees (Gakovic and Tetrick 2003). Because part-time employees value support and enablers more, this could also have a stronger impact in WFH.

### Empirical method

Results for means, standard deviations and correlations for all used variables are shown in Table 3. Due to item-nonresponses, the sample size used for the mediation analysis is 628 (*N*). This dataset from U.S. and German employees was used for all calculated models. Subsample analysis used samples from this dataset as well.

**Table 2:** Survey items and descriptive statistics

Items	M	SD	α
Work characteristics			0.921
• Scope for decision-making	5.09	1.41	
• Free time management	5.15	1.40	
• Autonomous decisions	5.15	1.40	
• Sequence of work	5.20	1.38	
• Number of various skills	5.39	1.25	
• Requirement for different skills per task	5.39	1.24	
• Number of tasks	5.31	1.29	
• Demanding skills	5.25	1.32	
• Skills variance	5.24	1.32	
• Work variety	5.18	1.34	
• Number of talents used	5.27	1.33	
• Frequency of new challenges	5.20	1.33	
Workforce			0.836
• Tolerance of other opinions	5.39	1.30	
• Change as part of the work	5.19	1.22	
• Team improvement	5.60	1.20	
• Take initiative	5.22	1.34	
• Foresighted way of working	4.86	1.47	
• Adjustment of work based on anticipated future development	5.25	1.20	
• Adaption as part of work	5.48	1.22	
• Mistake tolerant culture	5.58	1.22	
• Feedback	5.64	1.18	
• Mistakes as an opportunity to improve the work	4.89	1.42	
• Free expression of opinion	5.36	1.37	
• Stress resistance	3.59	1.09	
Organization			0.829
• Good collaboration	5.76	1.14	
• Shared responsibility	5.53	1.21	
• Importance of communication	5.55	0.170	
• Team decisions	5.15	1.26	
Work from home enabler			0.581
• Involvement in planning processes	5.22	1.34	
• Mutual trust	5.87	1.14	
• Trainings	5.08	1.42	
• Support availability	5.55	1.24	
• Technical availability	5.07	1.56	
Work from home success			0.890
• Satisfaction	5.51	1.32	
• Enjoyment of the work	5.61	1.32	
• Work from home preference	5.66	1.30	
• Increase in productivity	5.02	1.48	
• Facilitation of work	5.05	1.44	
• Quality improvement	4.87	1.47	

**Table 3:** Means, standard deviations and correlations

Variables		M	S.D.	1.	2.	3.	4.	5.
1.	WFH success	5.32	1.14	1				
2.	Agile work	5.21	0.75	0.383**	1			
3.	WFH enabler	5.38	0.82	0.383**	0.600**	1		
4.	Openness f. technology	4.67	0.76	0.253**	0.412**	0.299**	1	
5.	Age	36.82	10.47	0.156**	0.097*	0.073	− 0.093**	1

Notes: M = mean, SD = standard deviation and Pearson-Correlations (2-sided); **. * denotes significance at the 1% and 5% level, respectively

**Fig. 3:**
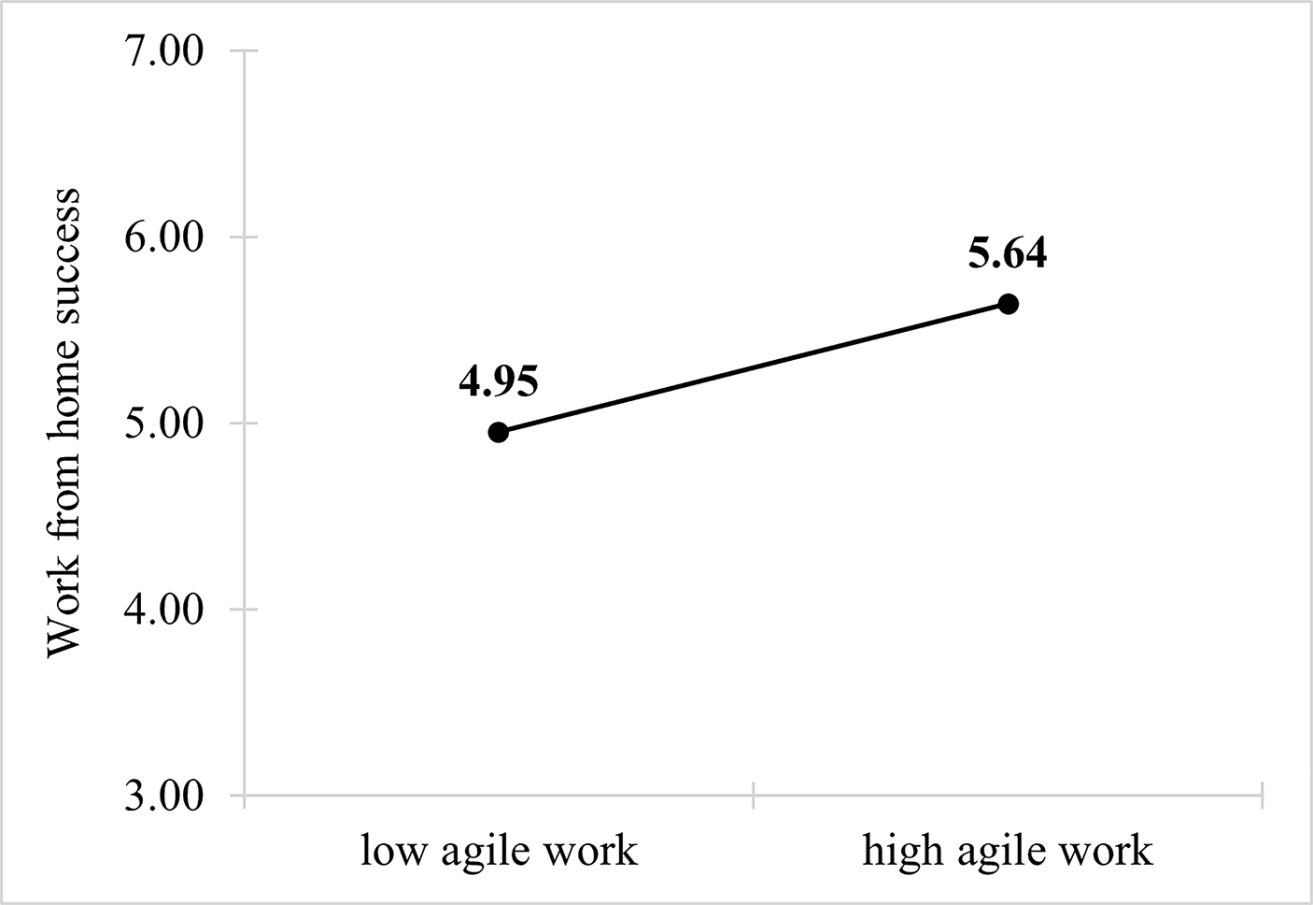
Mean value comparison of agile work and WFH success

Prior to the mediation analysis, a simple mean value comparison was performed to indicate a possible relation between the degree of agile work and WFH success. The presence of agile working practices can be seen in Fig. 3. If the degree of agile work is low, then the mean of WFH success is 4.95. If agile work is high, then the mean value of WFH success is 5.64. This possibly indicates an influence as derived from the literature.

The hypotheses were tested using various test procedures and regression analyses. The presented constructs of the independent and dependent variables, the control variables and the mediator were included in each regression model, and examined for mediation following Baron and Kenny (1986). Although the methodology still finds application in literature (e.g. Bendickson and Chandler 2019) there is criticism of the Baron and Kenny (1986) approach because the conclusions about mediation are not sufficiently meaningful and an assumption is made about the normality of the variable distribution (Zhao et al. 2010). Therefore, an alternative approach is recently used in the literature (e.g. Ghosh et al. 2017) based on a bootstrapping approach by Hayes (2009). Because of the possible shortcomings of the concept of Baron and Kenny (1986) the authors of this study followed this approach by using the PROCESS macro for SPSS by Hayes and Little (2018) (‘Model 4’ – simple mediation) to determine a possible mediated effect of agile work on WFH success through the WFH enabler. This approach uses ordinary least squares regression to determine un-standardised path coefficients of the direct, indirect and total effect.

We generated 10,000 bootstrap samples based on the observations derived from the 628 participants with heteroscedasticity-consistent standard errors in order to determine the confidence intervals (CI) and inferential statistics as proposed by Davidson et al. (1995). Effects were considered to be significant if 0 was not included in the confidence interval, following the approach by Hayes (2009).

## Results

A significant, positive effect of agile work on WFH success (*c*) was observed: *B* = 0.447, *p* < .001. The regression coefficient differs significantly from 0, which indicates a positive effect of agile work on WFH success. This confirms Hypothesis 1 (*A higher degree of agile work is related with higher WFH success*).

After entering the mediator and the control variables into the model, agile work predicted the mediator (*a*) significantly, *B* = 0.669, *p* < .001. This indicates a positive effect of agile work on the success of WFH enablers and supports Hypothesis 2 (*The more pronounced agile work is among employees, the more WFH enablers can be used*). WFH enabler predicted WFH success (*b*) significantly, *B* = 0.322, *p* < .001, which confirms Hypothesis 3 (*Employee support is positively associated with WFH success*) (Table 4).

**Table 4:** Results for hierarchical regression analyses

	Mediator: WFH enabler				Outcome: WFH success		
	*B*	*SE*	*p*-Value		*B*	*SE*	*p*-Value
Constant	1.418***	0.235	0.000		0.888**	0.336	0.008
OpennessTechnology	0.122***	0.038	0.001		0.174**	0.063	0.006
Gender	− 0.167**	0.056	0.003		− 0.046	0.087	0.598
Age	0.003	0.003	0.300		0.012***	0.004	0.001
Own workroom	0.069	0.056	0.216		0.338***	0.087	0.000
Leadership	− 0.091	0.066	0.169		0.038	0.101	0.708
Country US	− 0.167*	0.066	0.011		0.030	0.098	0.759
Agile work	0.669***	0.042	0.000		0.231**	0.084	0.006
WFH enabler					0.322***	0.072	0.000
adj-R²	0.394				0.223		

Notes: N = 628; *B*: Unstandardized regression coefficients; *SE*: robust standard errors; ***, ** and * denote significance at the 0.1%, 1% and 5% level, respectively

We found that the relationship between agile work and WFH success is partially mediated by WFH enablers, indirect effect *ab* = 0.216, 95%-CI[0.122;0.312] and direct effect *c*’ = 0.231, 95%-CI[0.065;0.397] (Table 5).

**Table 5:** Summary of the mediation effect of agile work using bootstrapping

Bootstrapping (10,000 Samples)	Direct effect	Indirect effect	Boot SE	95% confidence interval	
				LLCI	ULCI
Agile work → WFH enabler → WFH success	0.231***	0.216	0.048	0.122	0.312

Notes: N = 628; LLCI: lower level confidence interval; ULCI: upper level confidence interval; *SE*: robust standard errors; ***, ** and * denote significance at the 0.1%, 1% and 5% level, respectively; Effects were considered to be significant if 0 was not included in the confidence interval

We found significance for the control variable openness to digital technologies (*B* = 0.173; *p* < .01), stating that more openness to digital technologies is related with a higher WFH success. Also significant were age (*B* = 0.012; *p* < .01) and an own workroom (*B* = 0.338; *p* < .001). We find that older employees show higher values of work success when working from home. We found a significant effect of gender on the mediator WFH enabler (*B* = − 0.167; *p* < .01), indicating that female employees are more receptive for support measures. All other control variables, including country, have no significant effect on WFH success, which is in line with results of other studies (Bloom et al. 2015; Møller-Jensen et al. 2008).

**Fig. 4:**
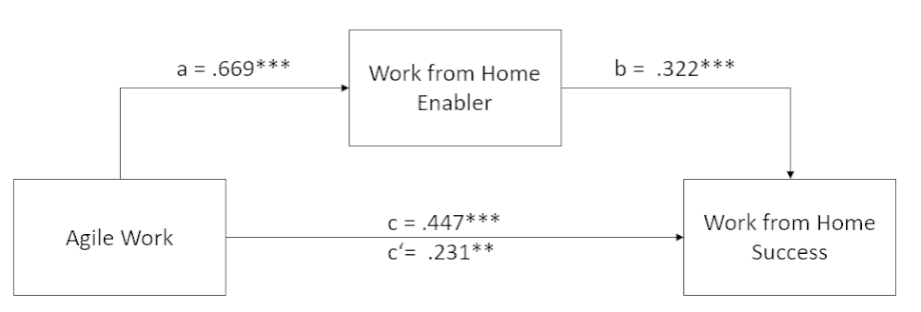
Results of the proposed research model. (***, ** and * denote significance at the 0.1%, 1% and 5% level, respectively)

In conclusion, an indirect effect of *ab* = 0.216 and a direct effect of *c*’ = 0.231 from agile work on WFH success can be significantly confirmed (cf. Figure 4). This leads to a total effect of *c* = 0.447. A partial mediation, accounting for 48.3% of the total effect, is confirmed with the significance of the indirect effect. There is empirical support for the partial mediation model on the effect of agile work on WFH success. Therefore, Hypothesis 4 (*The use of WFH enablers partially mediates the effect of agile work on WFH success*) can be confirmed.

In the subsample analyses, employees with high work experience (high: ≥ 10 years of work experience, *N* = 341) show effects comparable to the total: agile work has a significant, positive effect on WFH success (*c* = 0.445, *p* < .001; *a* = 0.657, *p* < .001; *b* = 0.243, *p* < 0.05; *c*’ = 0.276, *p* < .05; *ab* = 0.169, 95%-CI[0.032;0.313]). For employees with less work experience (< 10 years of work experience, *N* = 287), the effect of agile work on WFH success is no longer significant (*c*’ = 0.175, *p* > .1) after the mediator is included in the model. The total effect is mediated and explained by the use of WFH enablers (*a* = 0.659, *p* < .001; *b* = 0.425, *p* = .001; indirect effect *ab* = 0.281, 95%-CI[0.148;0.419]).

The second subgroup analysis provided similar effects for full-time employees (≥ 30 h per week, *N* = 505) as for the main study (* c* = 0.446, *p* < .001; *a* = 0.645, *p* < .001; *b* = 0.332, *p* < 0.05; *c*’ = 0.232, *p* < .01; *ab* = 0.232, 95%-CI[0.046;0.417]). The total effect for part-time employees (< 30 h per week, *N* = 123) is insignificant when the mediator is added to the model (*c* = 0.457, *p* > .1; *c*’ = 0.195, *p* < .05). The mediator effect is significant, indicating a total mediation (*a* = 0.759, *p* < .001; *b* = 0.346, *p* < .5, *ab* = 0.263, 95% CI[0.057;0.509].

## Discussion

This study proposes a new mediating relationship between agile work and WFH success. Further, the research framework defines and combines possible theories of agility, flexibility and adaptability of employees for WFH. Results indicate that companies with employees who have internalised agile working are, on the one hand, more successful in WFH and, on the other hand, can significantly support their employees through HRM actions. The use of WFH enablers has a partially mediating influence on the effect of agile work on WFH success. Thus, companies could positively influence the success of WFH in two ways: through the expansion of agile work and by applying WFH enablers. Agile work, therefore, is not only conducive to WFH success, but also allows HRM to deploy enablers in a particularly advantageous manner.

Agile work has a direct, positive and significant effect on WFH success. We show that characteristics and behaviours summarised under adaptive, flexible, dynamic or (as in this case) ‘agile’ work not only lead to an advantage in dynamic environments but are also specifically useful when working from home. Agile work can, therefore, help manage uncertainty during the COVID-19-pandemic and have a positive impact on employees’ work success and the firm’s outcome. We find that agile work is not only beneficial in dealing with the general uncertainty and dynamics of the COVID-19-crisis but also supports companies and has a positive impact on employees’ work success and the firm’s outcome. While leadership responsibility and the country have no significant influence, age and an own workroom significantly influence WFH success. As stated in other studies, an own workroom enhances productivity and, therefore, WFH success. Higher age corresponds to higher WFH success but cannot be assigned to a higher degree of agile work. Older employees may be more experienced and, therefore, less exposed to some of the challenges of WFH. Other (in this study unreported) descriptive statistics like the presence of children in the household or the sector of the workplace (industry) are not significant. The insignificance of industries is particularly noteworthy: provided the opportunity for WFH exists, this can be leveraged by employees in agile work regardless of industry. We controlled for possible differences between countries using a control variable but did not find a significant difference in WFH success.

Furthermore, Hypothesis 2 can be confirmed, which shows that agile work reveals potential for HRM support. This can be interpreted as follows. First, employees expect and value special kinds of support in terms of agile work. Freedom, participation, personal responsibility and trust by superiors are HRM measures to support agile employees. However, HRM should also place value on a culture in which mistakes are not seen as failures. This could have to do with the adaptive way agile employees work. With constant adaptation and development, mistakes can and perhaps must happen. Adaptation to change could also be complemented by change management to reduce resistance to change. At the same time, however, support, adequate technology and further training measures are also required. Gender influences enabler acceptance significantly: female employees tend to be more receptive to enablers offered. This illustrates further possible applications of enablers by HRM. Additionally, Hypothesis 3 was confirmed: support and enablement by management has a positive influence on WFH. Although employees are successful in WFH in terms of agile work, support is still essential. This effect, partially described in other, qualitative studies (Berube Kowalski and Swanson 2005), could also be observed quantitatively.

A partial mediation of WFH enabler on the relationship between agile work and WFH success was demonstrated, confirming Hypothesis 4. Thus, enablement by HRM at least partially explains the relation of agile work on WFH success and should, therefore, be extended. Analysis of the subsamples revealed the following: the work experience of the participants has an influence on the effects of the model. Experienced employees benefit from the direct effect of agile work and the mediated effect via WFH enablers when working at home. For inexperienced employees, the effects are different: the effect of agile work is entirely mediated by WFH enablers. This might be due to the needed experience for dealing with a high degree of freedom and self-structuring work tasks. Participants with less work experience have not gained the skills to derive a best-fit to cope with the high flexibility and autonomy. Enablers, therefore, have a dominant influence, especially because they can help build skills to deal with uncertainty and autonomy. Therefore, inexperienced colleagues should be especially supported by enablers in the sense of agile work. Training courses could build up targeted skills that enable the benefits of agile work.

Further, part-time employees value support from the organisation more than average and perceive it differently. In this respect, the results regarding the enabler are in line with the literature (Gakovic and Tetrick 2003). The positive perception of enablers could also strengthen the effect in the present case and make it particularly effective. Agile work has no direct, significant effect on WFH success in this subgroup. One reason could be that part-time employees often take up part-time work for specific reasons and, therefore, cannot benefit from autonomy and flexible, independent planning due to private reasons, for example. This could limit the direct effect of agile work. Another reason could be that part-time workers already make use of a high degree of flexibility due to their part-time work. In such, they demand a structured framework in their job and cannot value autonomy and freedom as high as full-time workers.

This study makes two main contributions. The first contribution is the investigation or further construction of agile work in a workplace context. As previously shown, countless definitions exist, some of which are not very clear-cut; at their core, they all deal with adaptability and flexibility in dynamic and uncertain situations. Even though essentially the model of Sherehiy and Karwowski (2014) was used as a basis, an adaptation was made and the new agile instrument can be used as a starting point for further construct validations. The framework was adapted and extended by including, e.g. participation and collaboration of the organisation in the sense of teamwork. Even though the original framework was mainly aimed at manufacturing, we received reasonable results for knowledge-intensive WFH. One could argue that the realisation that agile work is beneficial for WFH could be expected. However, it could also be argued that key aspects such as communication or collaboration could be more difficult due to the circumstances that could result from the isolation at home. Our study disproves this and emphasises the benefits of agile work for WFH. Although the ‘agile work’ construct used here does not aspire to cover the full range of potential flexible and adaptive capabilities, the selection served to illustrate potential characteristics of agile working. Even though CRA measurements fluctuated, agile workforce and collaboration could be identified as suitable constructs, especially relevant for employees with a high proportion of WFH.

The second contribution refers to possible support measures for WFH. For this purpose, a construct was defined that refers specifically to agile work. This represents a possibility to model support measures. An essential contribution is the modelling as a mediator. This assumes that there are measures that are particularly effective or can be used particularly successfully when employees work in the sense of agile work. In addition, the choice of the mediator model should be emphasised. Previous studies chose dynamic capabilities or flexibility as the mediating effect (Lin and Wu 2014; Wu et al. 2016). As suggested by other researchers, agile work as part of dynamic capabilities was not modelled as a mediator in this paper, but rather as a main predictor as it can be seen to be one of the main drivers of employee and firm success (Li and Liu 2014). The results show the great importance of agile work although, of course, there may be countless other effects on the success of WFH that were not part of this study. It turns out that these targeted measures are not only important in general, but also specific to employees in terms of agile work because a large proportion of the effect of agile work can be explained by the mediation effect.

Lastly, based on a relatively large sample in the U.S. and Europe, this study provides insights into data collected during the COVID-19-crisis and the lockdown with expansion of WFH. To our knowledge there are as for now no comparable studies with focus on capabilities with a similar topic.

### Practical implications

There are several possible practical implications for management and HRM in particular that arise from this study. On the one hand, the advantages of agile work that have been demonstrated make it necessary to control and expand these dynamic capabilities.

Work characteristics are closely related to the type of job and tasks. Measures to diversify tasks and changing roles could increase agile work. In order to be able to perform tasks with demanding and changing skills, training, experience and trial and error acceptance are essential. Central is the autonomy that can be granted to employees by superiors. Workforce as part of agile work is closely linked to employee behaviour and values. A role model of these values by managers and a suitable culture should be achieved and appropriate behaviour rewarded. Collaboration should be enabled as much as possible and demanded and communicated as a central success factor, especially in WFH. Participation should be offered on the one hand and demanded on the other. As we do also find support for openness to technology, we encourage employees to actively use offered enablers and training, and companies to provide this support by ensuring sufficient infrastructure. Companies can already inquire about openness with regard to digital technologies and their safe use during the hiring process. From the perspective of employees, they can point to existing experience and competencies in dealing with digital technologies and, thus, indirectly demonstrate their suitability for WFH or agile working.

Furthermore, the WFH enablers should be enhanced. These enablers should aim to actively involve employees despite the distance through participation in, e.g. planning, and to ensure mutual trust through a suitable culture and supervisor behaviour. Trust is particularly important to enable employees to plan and implement their work autonomously despite the distance and is a prerequisite; for example, for operating management by objectives without constantly monitoring the employee and his or her work progress. The subsample analysis revealed that inexperienced employees are even particularly dependent on these enablers because complete mediation exists. Here, specific measures and training can help make the best possible use of this effect. The subgroup of part-time employees is similar. They particularly appreciate and need support from the organisation. The use of enablers with a focus on agile work makes optimal use of this context. Special attention should be paid to technical infrastructure and support even though this may seem trivial. Without technical and professional support, employees of whatever ability are limited in their WFH role. Therefore, a suitable infrastructure, support and training concept should be implemented and constantly adapted. Furthermore, it must be noted that even if employees have great adaptive skills and flexibility to excel in WFH, the mediating function of the enablers must not be underestimated. In this respect, it is still necessary to provide or even expand support from the HRM perspective. Furthermore, the subsample analysis showed that inexperienced employees are even more dependent on support and that employees who work almost exclusively in WFH particularly participate in agile work. Both can be an opportunity to expand skills and measures to increase success in the WFH in a targeted manner.

The COVID-19-crisis has quickly demonstrated the importance of WFH in dynamic environments. This paper provides empirical insights into ways for HRM to respond to this: (1) by building relevant capabilities and (2) by aligning support and enablement. The emphatically simple model notes that these two measures go hand-in-hand and can be the answer to some of the problems associated with the crisis and the rapid increase in WFH. It is important to recognise that agile employees still need support for WFH; however, targeted support can be particularly successful and, therefore, seems to be very rewarding for HRM.

### Limitations and future work

There are few clear distinctions between partly synonymous or complementary constructs such as flexible workforce or adaptive capabilities. Based on existing studies, the authors of this study have strived to form constructs that are as meaningful as possible. However, these constructs are only a small part of theoretically possible capabilities that can be counted as ‘agile work’ as used in this study. Therefore, an extension by further or other aspects seems to be worthwhile. Further HRM measures to increase for instance self-efficacy and self-determination among employees can be included as it might also enhance WFH success. Even if the CRA measurements delivered satisfactory results, other constructs are conceivable. For example, an existing concept like ‘perceived organizational support’ (Eisenberger et al. 2001) could be examined as a mediator in the WFH setting. Furthermore, we interviewed employees who used WFH during the COVID-19-crisis. It can, therefore, be assumed that a large proportion did not voluntarily use WFH to such an extent. It is possible that results might differ if only voluntary WFH users were surveyed.

Conceptually, the choice of a mediator analysis seems very suitable. However, it would be interesting to examine control groups with little or no support or the selection of employees without attributes of agile work. A division of agile work into the three subcategories seems worthwhile to capture the derived findings and measures even more clearly. Likewise, additional mediators could be added, or the existing mediator could be further divided. These multi-mediator models could provide further detailed insights into the interrelationships. Therefore, a path analysis seems to be worthwhile because the sub-constructs are possibly further related to each other. In addition, the present model found different mediation throughout the model and subgroups. Therefore, in the future, further paths or subgroups should be analysed that map the relationship of agile work and WFH success in its entirety. Although there were no significant differences between the countries studied in this paper, other countries with different characteristics regarding WFH could be included in the future.

Even independent of the COVID-19-crisis, research predicts that the dynamic nature and uncertainty of the crisis will become more entrenched. In this respect, agile work is also essential regardless of this crisis - not least because it is assumed that WFH will continue to be maintained as there are advantages for employees, but also from sustainability aspects. In this respect, further research based on this research model is recommended to ensure success in the medium or long term. It should be noted, however, that this study primarily aims to provide a first step towards understanding agile capabilities in WFH.

## Electronic Supplementary Material

Below is the link to the electronic supplementary material.

Supplementary Material 1

## Data Availability

On request.

## Footnotes

1 We further checked average inter-item correlation for the construct WFH enabler, which has a value of 0.25 and is in an acceptable range.
