# Supplementary material for: Work from Home Success: Agile work characteristics and the Mediating Effect of supportive HRM
Source: Rev Manag Sci. 2022 Apr 12:1–26. Online ahead of print. doi: 10.1007/s11846-022-00545-5 (PMC9005023; doi:10.1007/s11846-022-00545-5)
Supplement: Supplementary file 1 — Supplementary Material 1 [file 11846_2022_545_MOESM1_ESM.docx]

Dear Reviewer,
Dear Editor,

First of all, thank you very much for the opportunity to submit a revised version and the constructive review and great suggestions. We found the comments very valuable and helpful and believe that the quality of the revised paper has improved with your support. We have especially revised the practical relevance sections to better highlight the significance of the study.

We have provided our responses to your individual comments in greater detail in the table below and hope that the revised paper will also be to your satisfaction.

**Note: The issues resulted from feedback from EURAM 2021 (Track Big Data, Data Analytics, and New Forms of Work), where this paper has been accepted.**

# Issues and responses

WFH = work from home

| **No.** | **Issue** | **C**hapter | **P**age | **L**ine | **Response** |
| --- | --- | --- | --- | --- | --- |
| 1-1 | A clear understanding of the purpose of the study has been presented with four hypotheses including an explanation for these hypotheses. This explanation looks repetitive throughout the introduction section, it can be reworded, concise, and summarized | Introduction | 2 - 4 |  | Thanks for this suggestion. Indeed, some content (e.g., benefits of agile work or possible concepts that enable flexibility/adaptability) was repetitive to the theory section. We limited the content of the introduction to the basics on a higher level and explain the benefits and concepts in detail in section 2. |
| 1-2 | The set of questions used for the survey is missing | Methodology | 12 | 318  and e.g.  249 | Thanks for the tip. We have tried to keep the article as readable and clear as possible and stay within the limited word count. Therefore, we have presented the question in short form in Table 2 (Survey items and descriptive statistics) and given one question per construct as an example in the derivation of the constructs (Measures section). We hope to do justice to the comment and at the same time keep the article compact. |
| 1-3 | Were all the three surveys were followed by the same set of people? | Methodology | 8 | 205 f. | Yes, the survey was aimed only at the same respondents, we matched the responses with a unique user key. That resulted in a pretty high drop-out rate, starting with 2415 respondents in the first wave, declining to 1016 respondents, taking part in all three waves. We included this information in the manuscript. (see also 1-5) |
| 1-4 | What were the selection criteria to pick people for the survey? | Methodology | 8 | 211  ff. | A prerequisite for participation in the survey was at least intermittent WFH employment. In the manuscript we now also elaborate on how the survey was administered (Lamapoll) and participants were contacted (Clickworker and Amazon's Mechanical Turk). Further, we rank results of such a survey design with reference to sources related to more traditional survey methods. |
| 1-5 | Were there any survey dropouts, if yes how many? | Methodology | 8 | 211 ff. | We provided detailed information about the participants: Out of 2415 participants starting with the first survey, 1016 people answered and finished also the third survey. This corresponds to a drop-out rate of 57.9%. This includes people who were excluded because their response time was too short. In addition, 28 respondents were excluded due to too short response time. |
| 1-6 | On page 19 second paragraph, the author mentions chapter 3 reference, without citation. Similarly, chapter 3 and 4 was mentioned on page 20 without citation | General | 14 | 410 | See 3-5, we removed references to other sections since it has led to some confusion. |
| 1 – 7 | In the limitation section, age can also a factor that could have impacted the study result. For example, the elder population working remotely might not be very comfortable using technologies and this could impact the word productivity | Limitations/ Measures | 11, 13 | 301 ff.  370 ff. | Thank you for this improvement. We now control for age and gender and find a positive significant relation for age, meaning that elderly can work more successful in the home office. This is in line with recent research, showing that older people are more productive in the home office as they do not have to rely on extensive communication, social interaction as younger people, as they already have built a larger network. We have also integrated more control variables for robustness and find significance for openness to digital technologies. |
| 2-1 | Additionally, I would have preferred to get insights into the theories you will contribute to. Is there a huge existing research stream, which you complement with your study, or is your study theory building by providing your results referring to agile work characteristics | Abstract | 2, 5 | 15 f., 101 ff. | There is a fast-growing research stream on dynamic capabilities and agility, on which we build on. Though, this has not yet been linked with a WFH setting. The underlying theory behind dynamic capabilities is the resource-based view of the firm. Employee agility as part of dynamic capabilities is regarded as an important resource of the firm. Recent literature shows, that this is a major success factor especially facing the COVID Crisis (Werder et al. 2020). We have now further developed this argument in our manuscript. |
| 2-2 | At the beginning of the introduction, I would have preferred to firstly address the cause for changes in the environment and corresponding threats (COVID-19 pandemic; new settings for companies; what distinguishes winners of the crisis from losers?), and then provide effects (companies have to rethink their working conditions, which forces a shift towards telework etc., which could have positive effects for the company) | Introduction | 2, 3 |  | Thanks for the tip. From our point of view, there are two ways to build this argument. We argue that it is a fundamental challenge for companies to be able to adapt and to ensure the survival of the company in the WFH environment. Only in the second step we add the COVID-19 crisis, which is an additional driver for insecurity and WFH. We have chosen this line of reasoning because we believe that our study also possesses relevance independent of COVID-19. The crisis is definitely an amplifier, but a change to WFH could also be expected, for example, for reasons of sustainability or an increase in insecurity due to international, economic dislocations or increasing digitalization. COVID-19 has accelerated or revealed this trend.  However, we also understand the proposed reasoning and are of course willing to adapt it, if preferred. |
| 2-3 | The second paragraph of your introduction could benefit from a clearer structure. The single sentences are not logically connected (you argue: 1. lack of research referring to telework and company success, 2. employees are expensive: what is the consequence? this expansion of working from home is a change for companies and employees: yes, but how is it connected to the fact that employees are an expensive resource?). | Introduction | 2 | 34 ff. | Thank you for this helpful hint. Indeed, especially the sentence "This is highly essential as personnel are the most expensive resource for firms" was logically impossible to classify. This referred to the resource-based importance of employees and their capabilities for the company. Even though this is correct, it is difficult to understand at this point. We have therefore removed this sentence and go into detail about the theories and meaning of agile work in the theory section. This makes the described paragraph of the introduction logically coherent. |
| 2-4 |  | Introduction |  |  | Merged with 2-3 |
| 2-5 | Furthermore, when you speak about uncertainty for employees, it would have been beneficial to know, which consequences could arise out of this uncertainty. E.g., if employees tend to show worse task performance, or consider leaving, then the negative impact for the company gets clearer. | Introduction | 2,3 | 36 ff. | The concept of "uncertainty" is indeed not fully clearly described. We added a description referring to perception of uncertainty (e.g., chaining and unclear requirements, uncertainty regarding new roles or expectations towards the employee). We further clarify the results of such an uncertainty: Employees with low tolerance for uncertainty are negatively affected which may lead to reduced well-being and distress (Smith et al. 2020). |
| 2-6 | You start the third paragraph with a very long topic sentence. I would recommend to start paragraphs with shorter sentences in order to make your hook for this paragraph and do not impair the reader’s understanding by wordiness | Introduction | 3 | 42 ff. | This is definitely true. We shortened the sentence significantly (see 2-7) |
| 2-7 | You speak about benefits for employees and companies, but the keywords you provide in this context doesn’t seem like benefits (e.g., psychological stress, family conflicts). I got your point, but in order to strengthen your statement, I would try to pick a clearer wording (avoidance of family conflicts if you speak about employees’ benefits or psychological “peace”). | Introduction | 3 | 42-45. | Indeed, we had an inconsistent logic in this (long) sentence. We sharpened the argumentation and pointed out clear benefits of WFH: The benefits of WFH for companies and employees have been studied, especially in terms of work equipment, productivity, work–life balance, flexible time allocation and reduced time loss due to fewer commuting (Gálvez et al. 2011; Nakrošienė et al. 2019) |
| 2-8 | You address important aspects within your introduction. I would recommend sharpening the potential threats from a company’s perspective that come up if they fail in adapt to these new conditions. This would give your contribution more value | Introduction | 3 | 39 ff. | We gladly clarified this: Without appropriate support for these challenges, employees are potentially affected by loneliness or loss of meaning, lower productivity and well-being, and companies are threatened in their ability to survive (Carnevale and Hatak 2020; Tremblay and Thomsin 2012) |
| 2-9 | When you speak about various approaches and theories referring to capabilities of a company and its employees on page 4, I would appreciate some exemplary studies (citation “e.g.,….” After the first sentence of this paragraph) | Introduction | 3 | 55 f. | We added reference to three central studies: Enterprise Agility, Dynamic Capabilities and Organizational Agility. The detailed explanation regarding these concepts were moved to the theory section (see 1-1) |
| 2-10 | You provide good explanations about the confusing and manifold definitions of agility. P. 5; second sentence: Again, I would recommend avoiding this sentence length since it entails unnecessary complexity | Introduction | 3 | 66 ff. | Thanks for this suggestion. The original sentence was too long to be easy to understand. We still want to list the manyfold benefits, but separated the sentences and changed the syntax:  “Agile capabilities and behaviours of employees are for example: participatory solution finding, willingness to change and adapt, tolerance of uncertainty, finding solutions through teamwork, or coping with challenging work through autonomy (Dyer and Shafer 2003; Griffin and Hesketh 2003; Samukadas and Sawhney 2004)” |
| 2-11 | Without in-depth knowledge, it is sometimes somewhat confusing to differentiate between HRM measures and Change Management measures. It would have been helpful to get a better understanding about the differences | Introduction | 4, 10 | 80 ff., 288 ff., | Thank you for this suggestion. Indeed, the use of HRM and change management is not separating enough. We have implemented this comment as follows: change management deals with the planned and structured accompaniment of change, with the goal of avoiding negative effects on employees ("resistances"). Approaches like participation and offering autonomy are HRM measures that also aim to empower employees towards change (Tummers et al. 2015). Parts of the change management construct are exactly these: participation in the planning process for WFH or trust in employees working in autonomy. We would therefore like to bring the enablers, which have so far been listed as "change management", under the group of HRM measures in order to achieve greater clarity regarding these measures on the one hand and to take into account the decisive role of HRM in change on the other. |
| 2-12 | However, when it comes to the development of the first hypothesis, the derivation is not clear: I understand the advantages of an agile work setting well, but I did not get, why these attributes (e.g., bidirectional communication, shared team responsibility) ultimately lead to a better work success from home | Theoretical Framework and Hypotheses | 6 | 133-147 | We have detailed the derivation of the first hypothesis: We have more clearly stated what benefits agile work in general has and elaborate why these characteristics are particularly suitable for WFH.   E.g.: Agile work is characterised by active bidirectional communication, proactivity and strong and shared team responsibilities (Hopp and Oyen 2004; Muduli 2013). Employees can communicate proactively or maintain team bonding, and at the same time have freedom in planning their work. This results in fewer interruptions while maintaining exchange of information despite distance to colleagues, which leads to an increase in productivity (Fonner and Roloff 2010) |
| 2-13 | In addition, I would recommend to formulate a proper Hypothesis: e.g., “A high degree of agile work contributes to a higher success in working from home Nevertheless, it might be hard to clarify, which extent of agile work means a high degree or extensive agile work. | Theoretical Framework and Hypotheses | 6, 7 | 149, 163, 177, 191 | Thank you for the suggestion. As correctly noted, it might be difficult to determine the exact degree of increase. Nevertheless, we have taken the comment as an opportunity to formulate directed hypotheses. |
| 2-14 | P. 9, second paragraph “However…”: There are very few citations. Are there no studies that addressed these relationships? | Theoretical Framework and Hypotheses | 6 | 151 | Thank you for this reference. We have clarified the list of possible benefits and provided sources:   However, agile work emphasizes skill variance, and high job demands (Sherehiy and Karwowski 2014) what may differ from other types of work and can therefore be challenging for employees not familiar with these work demands. This makes organizational support advisable. |
| 2-15 | Hypothesis 2: It might also be logical that employees who are not that used to agile work appreciate support in form of work from home enabler. | Theoretical Framework and Hypotheses | 6,7 | 151 – 164 | We agree that employees who are not used to agile work, particularly need support. However, a key issue is the type of support: We hypothesize that employees who are highly proficient in agile work are particularly used to be supported by special support measures. This is related to the requirements/characteristics of agile work, which are particularly receptive to this type of support through special measures (autonomy, support/trust from superiors, management by objectives).  We have clarified this argumentation in the manuscript and hope that it is to your satisfaction. |
| 2-16 | P. 10: Are these other studies (confirming a positive effect of work from home enablers on work from home success) also empirical ones? à potential reduction of your contribution? | Theoretical Framework and Hypotheses | 15 | 420 f | Comparable work is primarily qualitative research. We can therefore quantitatively substantiate these relationships based on quantitative, reliable regression analyses.  "This effect, partially described in other, qualitative studies (Berube Kowalski and Swanson 2005), could also be observed quantitatively." |
| 2-17 | Observing your summary of hypotheses, I asked myself “what’s behind the obvious?”. Maybe, it would be more helpful to tell companies, what are the most effective work from home enablers? Later I have seen that you have included different kinds of enablers in Table 2 à maybe this could strengthen your contribution additionally? | Theoretical Framework and Hypotheses |  |  | Thank you for this comment. We would love to contribute not only to theory, but also to practitioners. We therefore extended the practical implications and go into greater detail about the enablers and the individual items, that were measured with this construct. We have also extended the benefits from working agile and give advice, how to operationalize it. We hope that we have now enhanced the contribution. |
| 2-18 | How do you control for different capabilities referring to teleworking-tools depending on the different ages? This could also drive the need for support | Methodology | 11,  13,  14,  15 | 303 ff.  370 ff.  402 ff.  417 ff. | We have reworked the study design and integrated more control variables. We now control for age, gender, own workroom, leadership responsibility and openness to digital technologies. Only few of these controls are significant. The significance of the control variables and the interpretation of this effect are now included in “results” and “discussion”. For instance: We find significance for female employees for support measures. Own workroom is highly significant. These analyses are now integrated in the manuscript in detail and supplement our findings. |
| 2-19 | Why is Table 3 mentioned before Table 1? This makes no sense | Methodology |  |  | We have revised the numbering of the tables as well as their placement (see also 2-25). They now logically follow the references in the text. Furthermore, the tables were listed in the appendix, which could have led to misunderstandings (see 2-33). |
| 2-20 | Why did you focus on Germany and US? | Methodology | 11 | 311 ff. | Thank you for this comment. We have now added the reasoning in the manuscript. We wanted to compare the European work setting with the Anglo-Saxon work setting. Hence, we focused on Germany as one of Europeans country with the highest GDP and stable economic and political regulations. In order to compare the results with the Anglo-Saxon culture, we selected US as a country with high GDP, but differences in COVID-19 handling. Furthermore, WFH is more widespread in the USA than in Germany. This allows us to cover a greater variance (WFH while the COVID-19 crisis; first wave: US -> 42% (Bloom 2020) - Germany: 27% (Statista 2021) |
| 2-21 | Did you consider differences in political regulations referring to the pandemic? Germany forced a stricter risk-avoidance path than for example the U.S.; this, in turn, affects the working from home behaviour essentially. | Methodology | 11 | 311 ff. | As described, the share of workers in WFH varies between Germany and the United States. Even though Germany has reacted more strictly in terms of a lockdown, a higher proportion of employees in the USA have structurally switched to WFH. Therefore, we suspect that these effects offset each other. Please also see 2-20 |
| 2-22 | Additionally, I would have appreciated information about the industries they are working in. There might be industries, in which working from home might be better possible than in others. | Methodology | 14 | 407 | Thanks for this suggestion. Indeed, employees in different industries might also react differently to agile work or WFH. We added industries in table 1 for descriptive statistics. Further we tested industries as a control variable to account for possible effects. We found no significance in any regression. Therefore, we added this information in the discussion as unreported, insignificant control variable. If preferred, we could add this to the regression as well. |
| 2-23 | The distinction between work satisfaction and work productivity is positive. If working from home settings suit agile employees better, it is important to have a control group for the empirical examination (non-agile employees). Are there questionnaires that enable to detect the individual’s agility? This would help to increase validity of your study since there might be some kind of selection bias in your sample otherwise. | Methodology | 8 | 200 | In our study design, we did not limit the questionnaires to people working agile. The questionnaire was aimed at the population working (at least partially at home). We measured the three constructs work characteristics, workforce and organization, (that form the construct agile work) on 7-point likert scales. Therefore, we cover the whole variance of non agile work to full agile work. We have plotted the diagram, which we have included in this letter (Appendix – Fig 4). A control group would be possible, if there would be a natural experiment, e.g., if one company introduces agile working and we would measure before and after this event, which is not covered with our study design. |
| 2-24 | It does not become clear, what kind of measures are summarizes in “Change Management support”. | Methodology | 10 | 288 ff. | Analogous to note 2-11, we have adjusted the description of the enablers. Change management is an important part of HRM and is therefore summarized there. |
| 2-25 | Page 17: you refer to Table 1 in the text, and write “Put Table 2 about here”? | Methodology |  |  | We have revised the numbering of the tables as well as their placement (see also 2-19). They now logically follow the mentions in the text. Furthermore, the tables were listed in the appendix, which could have led to misunderstandings (see 2-33). |
| 2-26 | I was a little bit confused that in the results section are just correlation tables given, and not the regression tables. I expect to get deeper knowledge into the regression values, especially for example p-values instead of solely reporting the significance-level in a figure | Results | 13, 14 (Table 3 and Table 4) |  | Thank you for this suggestion. In the literature there are various possibilities to present results of a mediation analysis. We have now added the results for hierarchical regression analyses in another table and have chosen a graphical solution. We also added a summary of the mediation effect of agile work using bootstrapping presentation of direct and indirect effects.  We also put in the Process macro-output in the appendix of this letter. |
| 2-27 | Discussion: Since the discussion represents (besides the introduction) a major part of the paper, I would recommend not to start with “This study builds on other studies”: I would suggest to strengthen the first topic sentence of the introduction, and emphasizing the second part of this sentence | Discussion | 14 | 388 f. | Thanks for pointing it out, the wording and introduction of the discussion were poorly chosen. We have highlighted the contribution of the present study (mediation between agile work and WFH success). In our view, this serves a good readability and leads to the detailed discussion of the individual hypotheses. |
| 2-28 | The first finding (agile work creates more success in work from home) seems very intuitively | Discussion | 16 | 449 ff. | We can understand that this impression could arise. We have included a paragraph on this in the discussion. The statement "Agile work is beneficial for WFH" might seem trivial. Various components of agile work, such as communication or collaboration, are harder to achieve when working from home. Therefore, another way of reasoning could be that working together on-site (e.g.: in the company office) increases work success. However, we can show that agile working is nevertheless enhancing WFH success, for example via personal responsibility and proactivity, which makes this connection a little bit more non-trivial. |
| 2-29 | P. 19: I appreciate that you introduced the findings about work enablers. However, I cannot assign them to the support categories introduced before (Change Management support, HR measures etc.). | Discussion |  |  | Please see 2-10. |
| 2-30 | I think you can sharpen your findings by being clearer in the priority of measures/enablers. | Discussion |  |  | Thank you for your suggestion. We have reworked the practitioner contribution and reworked the description of the enablers and hope that it is to your satisfaction now. |
| 2-31 | I wondered why Work Satisfaction and Work Productivity are not included within the results, or at least how they affect Work from Home Success | Discussion | 11 | 295 ff. | As described on page 11, WFH success is composed of two constructs: Job satisfaction at home and work productivity at home. We have described in the "measures" section what is meant by these and which scales we used in the survey. Similar to the other constructs, we do not interpret the individual sub-constructs for the purposes of the survey. |
| 2-32 | Overall, I would recommend to properly checking the paper for spelling mistakes. I would recommend to be consistent in your spelling “work from home success” or “Work from Home Success”. | General |  |  | The wording of "work from home success", "work from home enabler", "agile work", etc. has been adapted or the abbreviation "WFH" used e.g. by Bloom et al. (2015) is used throughout the document |
| 2-33 | Furthermore, I would suggest cleaning up the Appendix in the document | General |  |  | We have completely cleaned up the appendix. The appendix was a bit cluttered when we handed it in because we followed the author instructions from Review of managerial Science when we handed it in to EURAM. We therefore put all the figures and tables at the end of the document and indicated in the text where each table/ figure should be. This probably caused confusion among the reviewers. |
| 2-34 | The methodology would benefit from greater explanation of the participants and procedures | Methodology | 8 |  | With the revision, we have better described the methodology (see also 1-3,1-4,1-5,2-20,2-21,3-1). We included descriptions about the participants, the procedure, regions, drop outs.  We further included Harman's single factor test, to rule out common method bias and find no such indication. |
| 3-1 | I am assuming the study population is the US or a region of the US? | Methodology | 8 | 205 ff. | See also 2-20. Yes, we combined German and US. The whole country of US was covered with respondents and also whole Germany. |
| 3-2 | Other than a reference to the longitudinal design, there is no explanation of how the participants were contacted by the authors, nor of the estimated response rate, if applicable | Methodology | 8 | 198 ff. | Thanks for this suggestion. We added information in the manuscript regarding the data sampling, the procedure, as well as response rate (42,1%). |
| 3-3 | However, as these variables have been largely adapted from other studies, I recommend that the authors undertake additional tests to demonstrate the reliability and validity of the adapted measures Of primary concern, the independent variables are positioned as multi-dimensional, untested constructs | Methodology |  |  | The variables used in our questionnaire, are derived from literature and partly adopted to the WFH setting. We first evaluated the items qualitatively in interviews and had them translated (the questionnaire was available in German and American English) by two independent translators and then evaluated again. We mostly relied on existing instruments, such as the Job Diagnostic Survey (Hackman and Oldham 1975), the Workforce Agility Scale (WAS) & Work Organization Scale (WOS) (Sherehiy and Karwowski 2014) and Work Design Scale (Stegmann et al. 2010). The content validity of the questionnaires was ensured through expert interviews from academia and industry. We also reported Cronbach’s Alpha which shows values above the threshold and has good reliability (except the construct WFH enabler). |
| 3-4 | The authors rightly point out the limitations of the Baron and Kenny (1986) test, and utilize Hayes' Process macro to test for mediation. Because the PROCESS macro is still relatively new, I recommend the inclusion of a brief explanation of what it is, along with the identification of the specific model that was used to test for mediation. | Methodology | 12 | 335 ff. | We added some clarification regarding the PROCESS macro. We used Model 4 of the Marco ("simple mediation"). Further we clarified the function of the macro: The use of ordinary least squares regression to determine unstandardized path coefficients of the direct, indirect and total effect. We further applied control variables in the analysis. |
| 3-5 | One small point, the authors refer to chapters in parts of the paper, which are likely directly derived from another paper, but need to be edited for this paper submission | General | general |  | We now avoid references to earlier sections in the paper. |

# Appendix

Fig. 1: PROCESS Mediation Output (1/3)

Fig. 2 PROCESS Mediation Output (2/3)

Fig. 3 PROCESS Mediation Output (3/3)

Fig. 4:Agile work variance

References

Berube Kowalski K, Swanson JA (2005) Critical success factors in developing teleworking programs. Benchmarking 12:236–249. https://doi.org/10.1108/14635770510600357

Bloom N (2020) How working from home works out. https://siepr.stanford.edu/research/publications/how-working-home-works-out. Accessed 10 January 2021

Bloom N, Liang J, Roberts J, Ying ZJ (2015) Does Working from Home Work? Evidence from a Chinese Experiment *. Q J Econ 130:165–218. https://doi.org/10.1093/qje/qju032

Carnevale JB, Hatak I (2020) Employee adjustment and well-being in the era of COVID-19: Implications for human resource management. J Bus Res 116:183–187. https://doi.org/10.1016/j.jbusres.2020.05.037

Dyer L, Shafer RA (2003) Dynamic organizations: Achieving marketplace and organizational agility with people

Fonner KL, Roloff ME (2010) Why Teleworkers are More Satisfied with Their Jobs than are Office-Based Workers: When Less Contact is Beneficial. Journal of Applied Communication Research 38:336–361. https://doi.org/10.1080/00909882.2010.513998

Gálvez A, Martínez MJ, Pérez C (2011) Telework and Work-Life Balance: Some Dimensions for Organisational Change. Journal of Workplace Rights 16:273–297. https://doi.org/10.2190/WR.16.3-4.b

Griffin B, Hesketh B (2003) Adaptable Behaviours for Successful Work and Career Adjustment. Australian Journal of Psychology 55:65–73. https://doi.org/10.1080/00049530412331312914

Hackman JR, Oldham GR (1975) Development of the Job Diagnostic Survey. Journal of Applied Psychology 60:159–170. https://doi.org/10.1037/h0076546

Hopp WJ, Oyen MP (2004) Agile workforce evaluation: a framework for cross-training and coordination. IIE Transactions 36:919–940. https://doi.org/10.1080/07408170490487759

Muduli A (2013) Workforce Agility: A Review of Literature. IUP Journal of Management Research 12

Nakrošienė A, Bučiūnienė I, Goštautaitė B (2019) Working from home: characteristics and outcomes of telework. IJM 40:87–101. https://doi.org/10.1108/IJM-07-2017-0172

Samukadas N, Sawhney R (2004) Workforce agility through employee involvement. IIE Transactions 36:1011–1021. https://doi.org/10.1080/07408170490500997

Sherehiy B, Karwowski W (2014) The relationship between work organization and workforce agility in small manufacturing enterprises. International Journal of Industrial Ergonomics 44:466–473. https://doi.org/10.1016/j.ergon.2014.01.002

Smith BM, Twohy AJ, Smith GS (2020) Psychological inflexibility and intolerance of uncertainty moderate the relationship between social isolation and mental health outcomes during COVID-19. J Contextual Behav Sci 18:162–174. https://doi.org/10.1016/j.jcbs.2020.09.005

Statista (2021) Anteil der im Homeoffice arbeitenden Beschäftigten in Deutschland vor und während der Corona-Pandemie 2020 und 2021. https://de.statista.com/statistik/daten/studie/1204173/umfrage/befragung-zur-homeoffice-nutzung-in-der-corona-pandemie/). Accessed 31 March 2021

Stegmann S, van Dick R, Ullrich J, Charalambous J, Menzel B, Egold N, Wu TT-C (2010) Der Work Design Questionnaire. Zeitschrift für Arbeits- und Organisationspsychologie A&O 54:1–28. https://doi.org/10.1026/0932-4089/a000002

Tremblay DG, Thomsin L (2012) Telework and mobile working: analysis of its benefits and drawbacks. IJWI 1:100. https://doi.org/10.1504/IJWI.2012.047995

Tummers L, Kruyen PM, Vijverberg DM, Voesenek TJ (2015) Connecting HRM and change management: the importance of proactivity and vitality. Journal of OrgChange Mgmt 28:627–640. https://doi.org/10.1108/JOCM-11-2013-0220

Werder K, Richter J, Hennel P, Dreesen T, Fischer M, Weingarth J (2020) A Three-pronged View on Organizational Agility. IT professional : technology solutions for the enterprise. https://doi.org/10.1109/MITP.2020.3016488
